# Efficient Detection of 2,6-Dinitrophenol with Silver Nanoparticle-Decorated Chitosan/SrSnO_3_ Nanocomposites by Differential Pulse Voltammetry

**DOI:** 10.3390/bios12110976

**Published:** 2022-11-06

**Authors:** M. Faisal, M. M. Alam, Jahir Ahmed, Abdullah M. Asiri, Mabkhoot Alsaiari, Raja Saad Alruwais, O. Madkhali, Mohammed M. Rahman, Farid A. Harraz

**Affiliations:** 1 Promising Centre for Sensors and Electronic Devices (PCSED), Advanced Materials and Nano-Research Centre, Najran University, Najran 11001, Saudi Arabia; 2 Department of Chemistry, Faculty of Science and Arts, Najran University, Najran 11001, Saudi Arabia; 3 Center of Excellence for Advanced Materials Research (CEAMR), King Abdelaziz University, Jeddah 21589, Saudi Arabia; 4 Department of Chemistry, Faculty of Science, King Abdulaziz University, Jeddah 21589, Saudi Arabia; 5 Department of Chemistry, Faculty of Science and Arts at Sharurah, Najran University, Najran 11001, Saudi Arabia; 6 Chemistry Department, Faculty of Science and Humanities, Shaqra University, Dawadmi 17472, Saudi Arabia; 7 Department of Physics, College of Science, Jazan University, Jazan 45142, Saudi Arabia

**Keywords:** 2,6-dinitrophenol sensor, differential pulse voltammetry, Ag-decorated Chitosan/SrSnO_3_ nanocomposites, ultrasonication, environmental safety

## Abstract

Herein, an ultra-sonication technique followed by a photoreduction technique was implemented to prepare silver nanoparticle-decorated Chitosan/SrSnO_3_ nanocomposites (Ag-decorated Chitosan/SrSnO_3_ NCs), and they were successively used as electron-sensing substrates coated on a glassy carbon electrode (GCE) for the development of a 2,6-dinitrophenol (2,6-DNP) efficient electrochemical sensor. The synthesized NCs were characterized in terms of morphology, surface composition, and optical properties using FESEM, TEM, HRTEM, BET, XRD, XPS, FTIR, and UV-vis analysis. Ag-decorated Chitosan/SrSnO_3_ NC/GCE fabricated with the conducting binder (PEDOT:PSS) was found to analyze 2,6-DNP in a wide detection range (LDR) of 1.5~13.5 µM by applying the differential pulse voltammetry (DPV) approach. The 2,6-DNP sensor parameters, such as sensitivity (54.032 µA µM^−1^ cm^−2^), limit of detection (LOD; 0.18 ± 0.01 µM), limit of quantification (LOQ; 0.545 µM) reproducibility, and response time, were found excellent and good results. Additionally, various environmental samples were analyzed and obtained reliable analytical results. Thus, it is the simplest way to develop a sensor probe with newly developed nanocomposite materials for analyzing the carcinogenic contaminants from the environmental effluents by electrochemical approach for the safety of environmental and healthcare fields in a broad scale.

## 1. Introduction

Generally, the toxic contamination of environmental water by organic pollutants is a great alarm to scientists. Among the toxins, nitrophenol and its derivatives are more toxic for the environmental and have a hazardous impact on human health [1]. Nitrophenols are comprehensively used raw materials in various industrial activities. In addition to this, they can be produced through the biodegradation of refineries and pharmaceutical and human wastes [2,3]. They are also directly used as raw chemicals to formulate pesticides, dyes, explosives, and pharmaceuticals to contribute to the world [4,5]. Therefore, there is a great probability for nitrophenols to contaminate land and water resources. Due to a lower degradation rate and high solubility, the exposure to low concentrations for a long duration in humans through the food chains can cause sycosis, cancer, and a malfunctioning of the liver, kidneys, and respiratory organs [6,7]. For good human physiology, it is very important to analyze nitrophenols, particularly 2,6-DNP in environmental water sources. For this purpose, existing methods, such as fluorescence, high-performance liquid and gas chromatography, spectrophotometry, and capillary electrophoresis are well-known [8,9]. However, these methods are limited by their various disadvantages, such as time-consuming nature, complicated process for analysis, expensive equipment, and less efficiency. Recently, the detection of such toxic phenolic chemicals is becoming popular by applying electrochemical methods including differential pulse voltammetry and others. This type of electrochemical detection is enhanced by modifying the anticipated working electrode with electron-sensing (metal-oxides/organometallic nanocomposites) substrates as reported previously [10,11]. Therefore, this study was directed toward the development of a 2,6-DNP electrochemical sensor applying Ag-decorated Chitosan/SrSnO_3_ NC/GCE as the electron-sensing mediators.

Up-to-date, various electron-sensing substrates, such as m-AgSAE [12], Cu–BTC/ERGO [13], Bi_2_O_3_ [14], and GO–MIP composites [15] have been successfully applied for the effective detection of 2,6-DNP using the DVP electrochemical approach. Among the organic materials, chitosan and its hybrid composites are well-known electron-sensing substrates in electrochemical applications. Therefore, chitosan composites, such as Cu–Chitosan/MWCNT NCs [16], Chitosan–Ag NPs [17], graphene–chitosan NCs [18], Chitosan–rGO/CILE NCs [19], and CPB–chitosan NCs [20] have been investigated for the detection of rutin, O-trihydroxy phenols, 2-NP and 4-NP, Bisphenol A, and dopamine and ascorbic acid, respectively. Therefore, chitosan composites have favorable electrochemical properties for the development of electrochemical sensors. The specificity of an enzyme-modified immunosorbent assay (ELISA) was modified by changing the chemical structure of the haptenized coating antigen. Then, a competitive indirect immunoassay was later developed, evaluated, and validated to measure target 2,4-DNP in wastewater samples. The assay used polyclonal antisera originally produced to analyze 4-nitrophenol, but by changing the chemical structure of the competitor, the resulting immunoassay was able to detect specifically and selectively 2,4-DNP by the ELISA technique [21]. A bifurcated optical fiber-based chemical sensor for continuous monitoring of 2,6-DNP was proposed based on the reversible chemical reaction between a novel functional poly(vinyl chloride) (PVC) as the sensing material and the analytes. The functional polyvinyl carbide containing a fluorescent curcumin moiety was synthesized by the nucleophilic substitution of a fraction of the chlorine atoms bound to the polyvinyl carbide backbone by curcumin [22]. As 2,6-DNP can provide optimal space geometry matches to the formation of hydrogen bonds, the sensor shows excellent selectivity for 2,6-DNP over other nitrophenols. A new approach for the detection of 2,4-dinitrophenol in solution is proposed here, which employs sensors based on silicon nanowire field-effect transistors with protective layers of high-*k* dielectrics, whose surface is functionalized with an amino silane [23]. Here, SOI-NW sensors were fabricated using CMOS-compatible technology, which allow a direct and highly sensitive detection of DNP with a 3  ×  10^−14^ M detection limit. These sensors can be used for the rapid monitoring of toxic compounds including DNP. These SOI-NW sensors are cheap, easy to use, and can be adapted for the detection of a wide range of toxic and explosive compounds by selecting sensor surface modification techniques.

In this study, Ag-decorated Chitosan/SrSnO_3_ NC/GCE was prepared by using the ultrasonic method and implemented as potential sensing substrates to analyze the selective 2,6-DNP in a phosphate buffer medium at a pH 7.0. As per the author’s knowledge, the developed Ag-decorated Chitosan/SrSnO_3_ nanocomposite has not been applied as an electron-sensing substrate for the development of the 2,6-DNP sensor by the electrochemical approach. Therefore, this study explored a detailed analysis of 2,6-DNP sensor parameters. In addition to this, the real samples were also electrochemically analyzed to test the fabricated sensor probe with reliability. The selective chemical sensing for the sensitive sensor development by using Ag-decorated Chitosan/SrSnO_3_ NC/GCE would be a prominent technique in the field of portable sensor technology for the efficient detection of unsafe compounds by an electrochemical approach in room conditions for the safety of environmental and healthcare fields in a broad scale.

## 2. Experimental Section

### 2.1. Materials and Methods 

In this approach, Sr(NO_3_)_2_, SnCl_4_·5H_2_O, Chitosan, AgNO_3_, NaOH, and CH_3_COOH were purchased from Sigma-Aldrich (USA) and used without further purification as received. For the characterization of Ag-decorated Chitosan/SrSnO_3_ NC/GCE, powder XRD investigations were conducted using a Bruker AXS-D4 Endeavour-X diffractometer (Radiation sources, CuKα_1/2;_ λα_1_ = 154.060 pm; λα_2_ = 154.439 pm). Shape and size and morphological- and surface-associated characterizations were made using a field-emission secondary electron microscope (FE-SEM) (JEOL-6300F, 5.0 kV) coupled with a high-resolution transmission electron microscope (HR-TEM) (JEOL JEM-2100F-UHR) operating at 200.0 kV with a built-in 1K-CCD camera and a Gatan GIF 2001 energy filter. For functional group characterization, FTIR analysis using KBr pellets in dispersion mode was performed with a Perkin Elmer Raman Station 400 spectrometer (range from 400 to 4000 cm^−1^). A VGESCALAB 200R spectrometer fitted with an MgKα (hν = 1253.6 eV) nonmonochromatic X-ray source and a hemispherical electron analyzer was used to perform X-ray photoelectron spectroscopy (XPS) analysis. During the initial treatment process, freshly prepared samples were degassed before they were shuffled in an ultra-high vacuum analysis chamber for 1 h. A Quantachrome NOVA 4200 analyzer was used at a temperature of 77.0 K to acquire the nitrogen adsorption isotherm for surface analysis. Each sample was sufficiently degassed at 200.0 °C overnight. The prepared nanocomposite surface area was efficiently measured via the Brunauer–Emmett–Teller (BET) technique using adsorption data in this approach. Later, diffuse reflectance spectra in a range from 200 to 800 nm were obtained on a UV-vis spectrophotometer (Shimadzu: UV-3600 plus). Electrochemical analysis for sensor application was performed with auto-lab potentiostat (Metrohm-Autolab modules) using three-electrode system for the development of sensor probe with Ag-decorated Chitosan/SrSnO_3_ NC/GCE in room conditions.

### 2.2. Preparation of SrSnO_3_

Perovskite SrSnO_3_ nanomaterial was prepared by a facile two-stage process as reported earlier [24]. Typically, 10.0 mmol of Sr(NO_3_)_2_ and 10.0 mmol of SnCl_4_·5H_2_O were pulverized to fine powders separately in an agate mortar. These powders were then mixed collectively and instantaneously, and 70.0 mmol of NaOH was then added to this mixture. Vigorous grinding of the formed mixture was continued for 1 h. During the grinding procedure, white smoke and a substantial amount of heat evolved. Attained powder of nanomaterial was then washed thoroughly with distilled water several times and later fully dried at 65.0 °C for overnight. The acquired powder of nanocomposite was later calcined at 750.0 °C for 2 h at 2.0 °C/min heating rate to achieve the desired perovskite SrSnO_3_ nanostructure.

### 2.3. Preparation of Ag-Decorated Chitosan/SrSnO_3_ NCs

Here, a very simple ultra-sonication approach was utilized to synthesize 10% Chitosan/SrSnO_3_ nanocomposites. Typically, 2.0 gm of SrSnO_3_ and 0.2 gm of Chitosan were ultrasonicated together for 30 min in 100 mL double-distilled water. After filtration, the as-obtained mixture was thoroughly washed 3/4 times with distilled water and ethanol and then dried in an oven at 65 °C for 24 h to obtain 10% Chitosan/SrSnO_3_. One percent Ag nanoparticles–10% Chitosan/SrSnO_3_ was synthesized by photoreduction technique as previously reported [25]. Briefly, 0.5 g of 10% Chitosan/SrSnO_3_ material was mixed with 100.0 mL of AgNO_3_ solution consisting of the required quantity of Ag^+^ to acquire 1% Ag concentration in the composite framework. The above suspension was continuously stirred for 12 h under UV light irradiation. Later, the as-synthesized material was collected and centrifuged. Finally, nanocomposite was washed with water and acetone simultaneously three times. The as-obtained product was dried at 65 °C for 24 h in order to obtain the required Ag-decorated Chitosan/SrSnO_3_ NCs. This nanocomposite material was used for total characterization by XRD, HR-TEM, FESEM, EDS, XPS, FTIR, and UV-visible spectroscopy and applied for the sensor applications with three-electrode system.

### 2.4. Fabrication of GCE Using Ag-Decorated Chitosan/SrSnO_3_ NCs

Modification of GCE to an anticipated working electrode is a critical task in the sensor probe development. To do this, the prepared Ag-decorated Chitosan/SrSnO_3_ NCs (0.5 µg) were used to make a slurry in ethanol. Later they were used to coat the GCE surface in a way that yielded a uniform thin layer of NCs onto the GCE. A few drops of PEDOT:PSS were added to serve as the polymer mixture on the GCE. After drying, the modified GCE, acting as the working electrode, was supplemented with Metrohm-Autolab modules at a parallel connection with an Ag/AgCl(Saturated KCl) and a Pt wire, used as a reference and a counter electrode, respectively. The target analyte (2,6-DNP) was analyzed electro-chemically in a concentration range of 1.5~13.5 µM. The obtained data (e.g., sensitivity, response time, LOD, LOQ, reproducibility, and LDR) were evaluated. Finally, the real samples contaminated with 2,6-DNP were measured. After using the NC-fabricated GCE electrode, it was cleaned with the DI water first and then ethanol in a beaker for 5 min. Then, the beaker was put into the ultrasonicator for 10 min to clean the electrode surface properly. By ultrasonication, the physisorbed and chemisorbed materials were removed significantly. Before fabricating the electrode for further electrochemical investigation, it was electrochemically cleaned in 0.5 M H_2_SO_4_ for 10 cycles by cyclic voltammetry using potetiostat at a scan rate (0.1 V/s).

## 3. Results and Discussion

### 3.1. Physical Characterization of Ag-Decorated Chitosan/SrSnO_3_ NCs 

**Figure 1:**
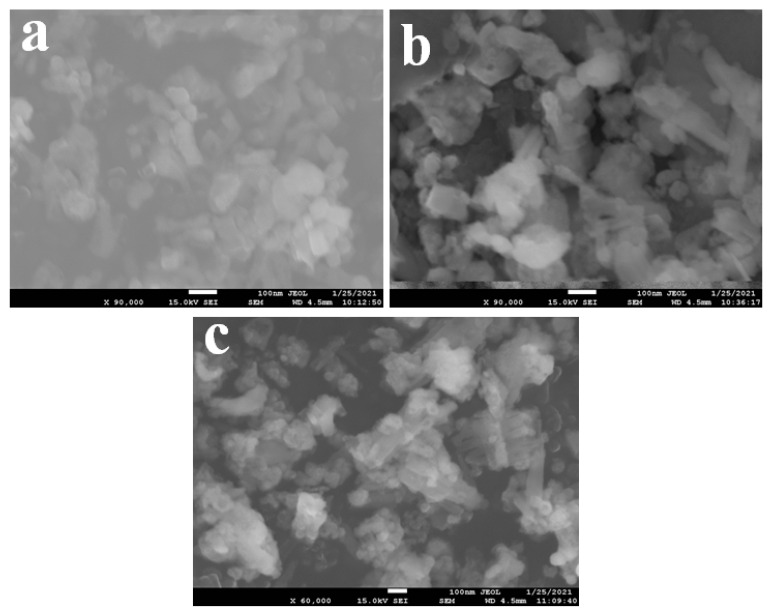
FESEM analysis: (**a**) pure SrSnO_3_, (**b**) Chitosan/SrSnO_3_, and (**c**) Ag-decorated Chitosan/SrSnO_3_ NCs.

Figure 1 represents the FESEM analysis of the Ag-decorated Chitosan/SrSnO_3_ NCs. As presented in Figure 1a, the shape of the pure SrSnO_3_ exhibits an irregular structure. When it was composited with Chitosan, the prepared SrSnO_3_ particles were adsorbed onto the surface and formed a shape without any structure, as demonstrated in Figure 1b. After the addition of the Ag nanoparticles by photoreduction, they were also deposited onto the surface of the Chitosan/SrSnO_3_ NCs, resulting in a nanomaterial without a particular morphology. Thus, Ag-decorated Chitosan/SrSnO_3_ is considered a nanocomposite (NC). 

**Figure 2:**
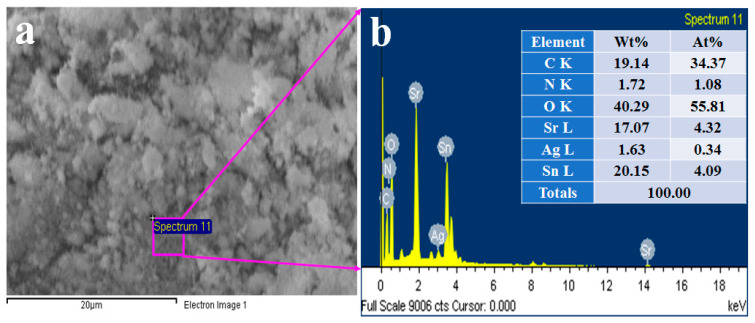
EDS analysis of Ag-decorated Chitosan/SrSnO_3_ NC. (**a**) Higher magnifying image and (**b**) Elemental composition of synthesized Ag-decorated Chitosan/SrSnO_3_ NC.

Analogous to Figure 1, a highly magnified EDS image is shown in Figure 2a. The elemental analysis obtained by EDS (selected area) confirms the existence of 19.14% C, 1.72% N, 40.29% O, 17.07% Sr, 1.63% Ag, and 20.15% Sn presented in Figure 2b. Therefore, the EDS analysis provides evidence of the existence of C, N, O, Sr, Ag, and Sn in the prepared Ag-decorated Chitosan/SrSnO_3_ NC. 

**Figure 3:**
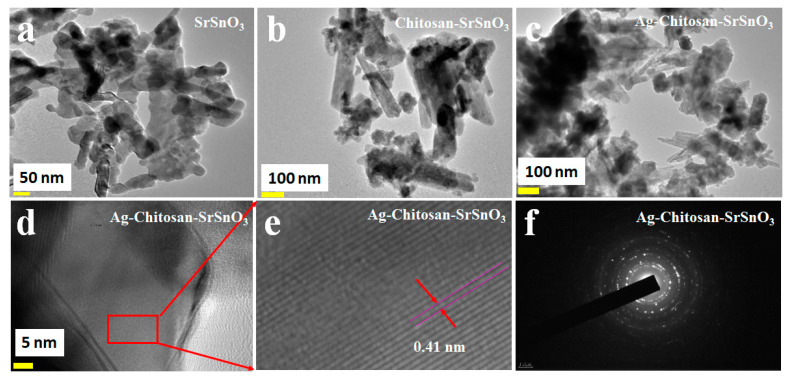
HRTEM analysis of Ag-decorated Chitosan/SrSnO_3_ NC. (**a**) SrSnO_3_, (**b**) Chitosan/SrSnO_3_, (**c**–**e**) low-to-high magnified Ag-decorated Chitosan/SrSnO_3_ NC, and (**f**) SAED pattern of NC.

For a detailed morphological analysis of Ag-decorated Chitosan/SrSnO_3_ NCs, HRTEM was employed to confirm their morphological structure. The obtained images are presented in Figure 3. Figure 3a shows the irregular nanoparticle structure of SrSnO_3_. Similarly, Figure 3b–d show the irregular adsorption of SrSnO_3_, Chitosan/SrSnO_3_, and Ag–Chitosan/SrSnO_3_, not forming any shape. From the magnified HR-TEM images, the lattice spacing was calculated as 0.41 nm (Figure 3e), and the SAED is given in Figure 3f.

**Figure 4:**
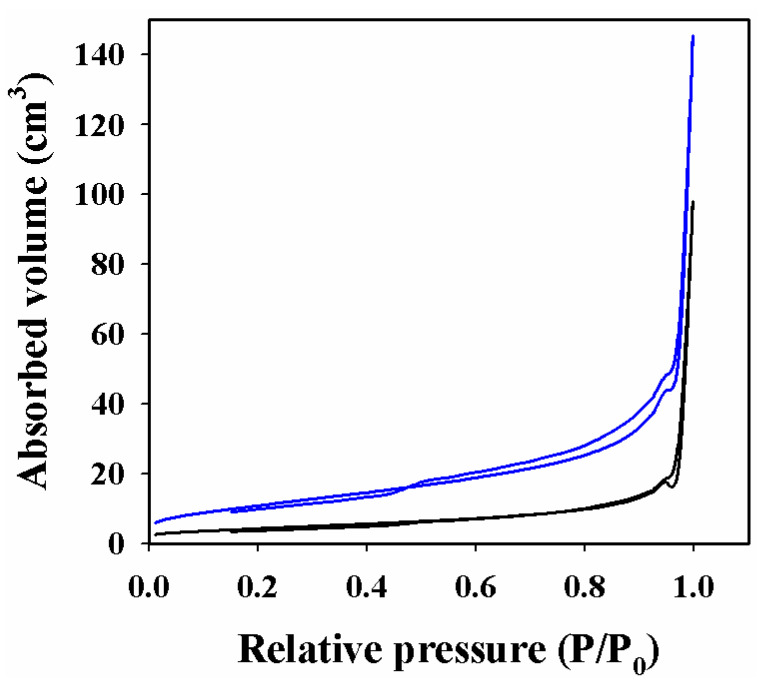
BET analysis of (black line) SrSnO_3_ and (blue line) Ag–Chitosan/SrSnO_3_ NCs. Black line refers to the BET results of SrSnO_3_. Blue line refers to the BET results of Ag-Chitosan/SrSnO_3_ NCs.

The active surface area was measured for the Ag-decorated Chitosan/SrSnO_3_ NC material and presented in Figure 4. Here, the surface characteristics of the prepared Ag-decorated Chitosan/SrSnO_3_ NCs are clarified through a nitrogen adsorption/desorption isotherm, which is known as a BET analysis. A plot of the relative pressure versus the adsorption of nitrogen gas was used to calculate the relative surface area of SrSnO_3_ (black line) and Ag-decorated Chitosan/SrSnO_3_ NCs (blue line) as 14.3 and 39.7 m^2^/g, respectively. Therefore, the morphological and textural studies of the Ag-decorated Chitosan/SrSnO_3_ NCs showed values favorable to exhibit an enhanced electrocatalytic performance.

### 3.2. Surface Composition by XPS Analysis

**Figure 5:**
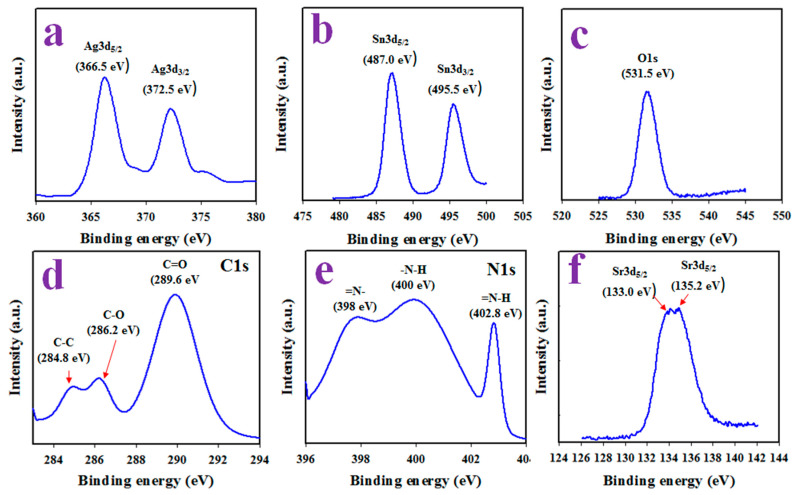
XPS analysis of Ag-decorated Chitosan/SrSnO_3_ NC, (**a**) Ag3d, (**b**) Sn3d, (**c**) O1s, (**d**) C1s, (**e**) N1s, and (**f**) Sr3d spectrum.

An XPS analysis of the Ag-decorated Chitosan/SrSnO_3_ NC was performed to identify the oxidation states of the individual constituting atoms, as demonstrated in Figure 5. As displayed in Figure 5a, the Ag3d orbital exhibits two spin orbitals Ag3d_5/2_ and Ag3d_3/2_, were positioned at 366.5 and 372.5 eV, respectively. The 6.0 eV energy difference between these two spin orbitals confirmed the existence of the Ag^+^ oxidation state in the prepared Ag-decorated Chitosan/SrSnO_3_ NC [26,27]. The XPS spectrum of Sn3d_5/2_ and Sn3d_3/2_ was fitted with the binding energies of 487.0 and 495.5 eV, respectively, as shown in Figure 5b, which has been found for Sn^4+^ ionization, as reported elsewhere [28,29]. As explored in the Figure 5c, O1s shows a peak positioned at 531.5 and confirmed the oxygen (II) oxidation state [30]. As presented in Figure 5d, carbon is a constituting element of this Ag-decorated Chitosan/SrSnO_3_ NC, and the C1s orbital is illustrated in Figure 5d and contains C–C, C–O, and O=C–O bonds, which are positioned at 284.8, 286.2, and 289.6 eV, respectively, in accordance with results reported elsewhere [31,32]. In addition to this, nitrogen is another constituting element of Ag-decorated Chitosan/SrSnO_3_ NC, and the N1s orbital is explored in Figure 5e, representing the N1s orbital. As shown, the three bonds of nitrogen appeared at 398.0, 400.0, and 402.8 eV corresponding to -N–H_2_, -N–H, and =N–H^+^, respectively [33,34]. In addition, the Sr3d orbital in Figure 5f shows two spin orbitals located at 133.0 and 135.2 eV and provides the evidence of Sr^2+^ oxidation [35,36]. 

### 3.3. Optical and Structural Characterization of Ag-Decorated Chitosan/SrSnO_3_ NCs

**Figure 6:**
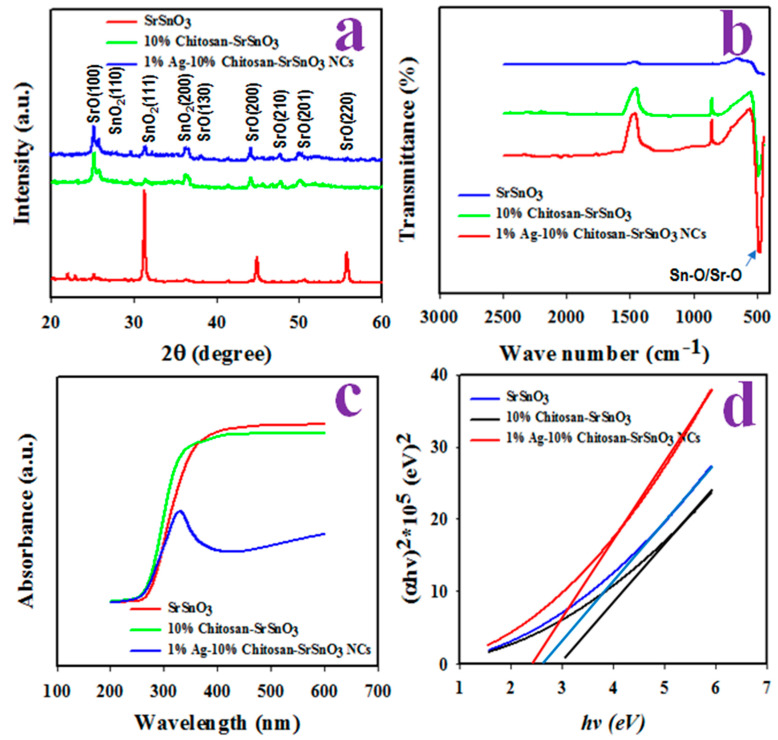
Structural and optical characterizations of Ag-decorated Chitosan/SrSnO_3_ NCs: (**a**) Powder XRD pattern, (**b**) FTIR, and (**c**,**d**) UV-vis optical absorbance.

A crystallographic analysis of the synthesized NCs of the Ag-decorated Chitosan/SrSnO_3_ was performed as illustrated in Figure 6a. The XRD pattern exhibits the crystalline planes of SnO_2_ at (110), (111), and (200) and identified in previous reports [37,38]. In addition to this, the crystalline plans of SrO, such as (100), (130), (210), (201), and (220), are also identified [39,40]. The grain size of the crystalline plan was calculated from Scherer’s equation (Equation (1)) and obtained to be 19.2 nm as calculated from the SrO (111) planes.

D = 0.9 λ/(βcosθ)(1)

Here, λ = X-ray wavelength in 1.5418 Å; β = full width measured at half (FWHM) of the peak in at diffracted angle θ.

The functional groups of the Ag-decorated Chitosan/SrSnO_3_ NCs were subject to FTIR investigation, as illustrated in Figure 6b. A peak was obtained at 500 cm^−1^, identified as the stretching vibration of the Sn–O bond [41,42]. Using Tauc’s equation, the optical bandgap energy of the Ag-decorated Chitosan/SrSnO_3_ NCs was calculated from the UV-visible absorbance, as presented in Figure 6c,d. As measured, the optical bandgap energy of the Ag-decorated Chitosan/SrSnO_3_ NC is equal to 2.5 eV, which is less than the bandgap of the Chitosan/SrSnO_3_ NC (2.8 eV) and pure SrSnO_3_ (3.0 eV). Therefore, the prepared Ag-decorated Chitosan/SrSnO_3_ NCs have more conductivity compared to either Chitosan/SrSnO_3_ NCs or pure SrSnO_3_.

### 3.4. Electrochemical Characterization of Ag-Decorated Chitosan/SrSnO_3_ NC/GCE

**Figure 7:**
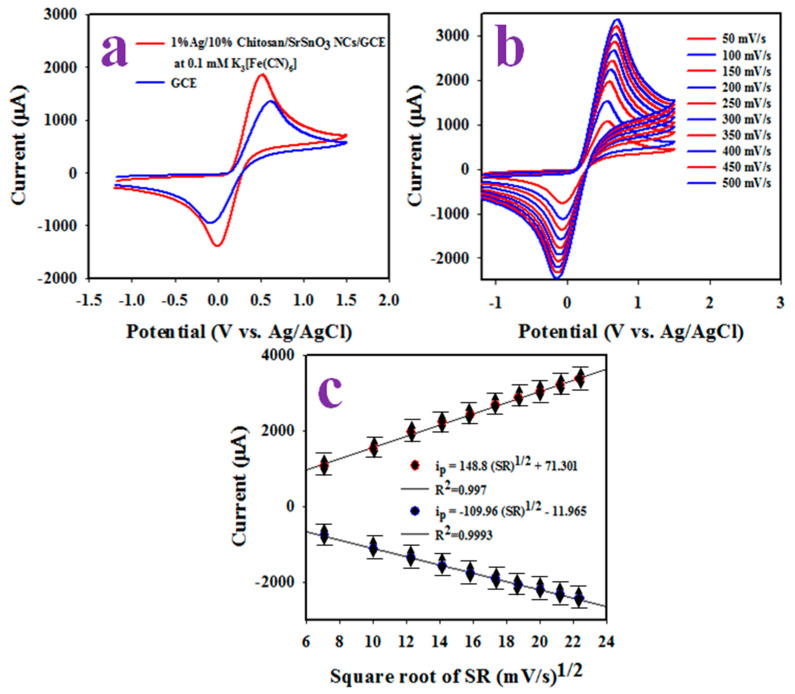
Electrochemical characterization of Ag-decorated Chitosan/SrSnO_3_ NC/GCE by cyclic voltammetry. (**a**) Comparison of coated and bare GCE in analysis of 0.1 mM K_4_[Fe(CN)_6_], (**b**) Scan rate at 50~300 mV/s, and (**c**) Current versus square root of SR.

For the voltammetric characterization of the prepared Ag-decorated Chitosan/SrSnO_3_ NC/GCE, 0.1 mM K_4_[Fe(CN)_6_] redox couple was used, as shown in Figure 7a. The potential difference between the oxidation and the reduction peak current of the coated GCE was +0.40 V compared to bare GCE (+0.70 V), which is higher than that of the Ag-decorated Chitosan/SrSnO_3_ NC/GCE. Therefore, the working electrode based on the Ag-decorated Chitosan/SrSnO_3_ NC/GCE has a higher current response, favorable to the electrochemical analysis of an analyte. Similar observations have been described elsewhere [43,44]. To evaluate the molecular diffusion on the surface of the anticipated working electrode, a plot showing the current versus the square root of the scan rate at a range of 50~300 mV/s is shown in Figure 7c. The experiment was conducted using 0.1 mM K_4_[Fe(CN)_6_]. The resulting peak currents are linearly distributed on the lines in both oxidation and reduction and can be expressed by the following equations (Equations (2) and (3)).

i_p_ = 148.8 (SR)^1/2^ + 71.301; R^2^ = 0.997 at oxidation of K_4_[Fe(CN)_6_] (2)

i_p_ =−109.96 (SR) − 11.965; R^2^ = 0.9993 at reduction of K_3_[Fe(CN)_6_] (3)

The above two equations confirm the good linearity and that the rate of reactions is a diffusion-controlled rate of molecules on the working electrode surface, which has also been shown by previous authors [45].

### 3.5. Detection of 2,6-DNP with Ag-Decorated Chitosan/SrSnO_3_ NC/GCE

**Figure 8:**
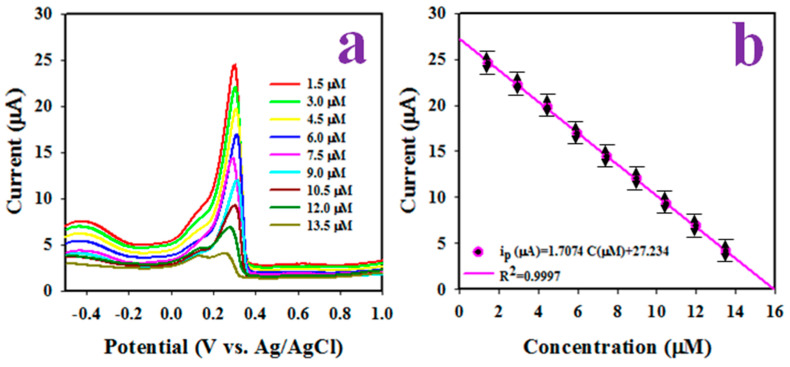
Detection of target analyte with Ag-decorated Chitosan/SrSnO_3_ NC/GCE. (**a**) Resulting DPV analysis of 2,6-DNP at 7.0 pH buffer and (**b**) Calibration of 2,6-DNP sensor obtained from current versus concentration of 2,6-DNP.

In this approach, differential pulse voltammetry (DPV) is a potential electrochemical method for analyzing an analyte. Therefore, 2,6-DNP in a buffer medium of pH 7.0 was subject to a DPV analysis based on a concentration range of 1.5~13.5 µM, as presented in Figure 8a. The intensity of the peak current decreased as the concentration of 2,6-DNP increased. Thus, the peak current points are plotted against the corresponding concentrations of 2,6-DNP in Figure 8b. The obtained current is distributed in a linear manner at a 2,6-DNP concentration range of 1.5~13.5 µM, which is denoted as the linear detection range (LDR) of 2,6-DNP for the assembled electrochemical sensor. Sensor parameters, such as sensitivity (54.032 µA µM^−1^ cm^−2^), were calculated from the slope of the LDR (1.7074 µA µM^−1^) by considering the active surface area of the GCE (0.0316 cm^2^). The LOD was estimated as 0.18 ± 0.0088 µM from the 3SD/σ, where SD refers to the standard deviation of a blank response, and σ refers to the slope of the calibration curve. LOQ is also calculated as 0.545 µM, from the equation of LOQ = 10 LOD/3.3. The linear equation of LDR can be expressed by Equation (4) as follows:

i_p_ = 1.7074 C(µM) + 27.234; R = 0.9997 at a reduction of 2,6-DNP (4)

**Figure 9:**
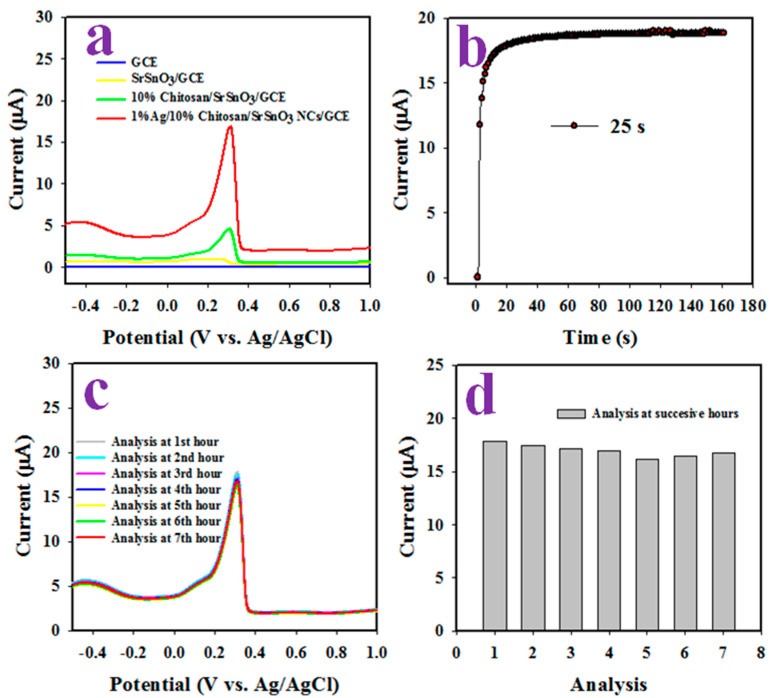
Analysis of sensor performance with Ag-decorated Chitosan/SrSnO_3_ NC/GCE. (**a**) Control experiment in DPV analysis of 2,6-DNP, (**b**) Response time at current versus time, (**c**) Reproducibility of 2,6-DNP sensor, and (**d**) Reproducibility bar diagram.

A control experiment was performed, as illustrated in Figure 9a, to evaluate the reduction performance of 2,6-DNP by the constituting elements of the prepared Ag-decorated Chitosan/SrSnO_3_ NC/GCE, such as SrSnO_3_, Chitosan/SrSnO_3_, and Ag-decorated Chitosan/SrSnO_3_ NC-coated GCE. The GCE modified by Ag-decorated Chitosan/SrSnO_3_ has the highest peak current, and the bare GCE has a zero-peak current. Thus, for the 2,6-DNP analysis, Ag-decorated Chitosan/SrSnO_3_ NC/GCE reasonably improved the conductivity of the anticipated working electrode via the DPV method. To specify the response of the 2,6-DNP sensor, 6.0 µM of 2,6-DNP was subjected to analysis, and the results are plotted as current versus time in Figure 9b. The current responses became steady at 25 s. Thus, 25 s was considered the response time of the 2,6-DNP sensor for the electrochemical analysis. 

The reproducibility of an electrochemical sensor is an important parameter that provides reliability information about it. Thus, the reproducibility test was executed by reaping seven analyses of 2,6-DNP at 6.0 µM in a phosphate buffer medium of pH 7.0 using the same working electrode as shown in Figure 9c and a bare diagram in Figure 9d. As shown, the intensity values of the peak currents in the reduction of 2,6-DNP are completely similar and cannot be separated. Thus, this test confirms that the assembled sensor based on the Ag-decorated Chitosan/SrSnO_3_ NC/GCE has good reliability for analyzing 2,6-DNP. Comparison studies [46,47,48,49,50] of 2,6-DNP detection based on various nanocomposites sensing electrodes are demonstrated and included in the Table 1.

**Scheme 1:**
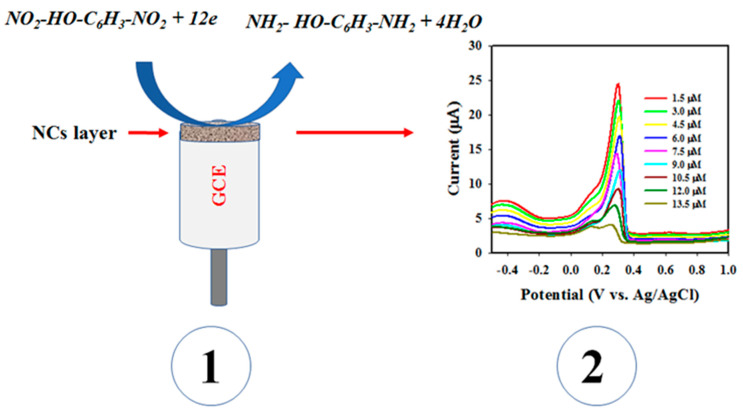
Schematic representation of chemical sensing with Ag-decorated Chitosan/SrSnO_3_ NC/GCE. (**1**) Electrochemical reduction of 2,6-DNP and (**2**) Concentration-based responses of 2,6-DNP to 2,6-DAP.

As shown in Table 1, the 2,6-DNP sensor based on the GCE modified by Ag-decorated Chitosan/SrSnO_3_ NCs exhibited improved performances in terms of sensitivity, LOQ, and LDR. The electrochemical quantification of 2,6-DNP based on the Ag-decorated Chitosan/SrSnO_3_ NC/GCE sensor is presented in Scheme 1. As shown, 2,6-DNP is adsorbed on the surface of the modified GCE. Due to the applied potential, it is reduced to 2,6-diaminophenol, indicated in the reaction scheme below. Comparable electrochemical reduction reactions of 2,6-DNP are reported in other articles [51,52,53,54]. The suggested electrochemical reaction of 2,6-DNP on the Ag-decorated Chitosan/SrSnO_3_ NC/GCE surface is as follows (Equation (5)):

NO_2_ − HO − C_6_H_3_ − NO_2_ + 12e^−^ → NH_2_ − HO − C_6_H_3_ − NH_2_ + 4H_2_O (5)

Finally, the NC-assembled sensor was used for the electrochemical analysis of real environmental samples, which were collected from distinct environmental sources, including underground water, tap water, and sea water. This analysis was performed by applying the standard addition method. The concentration of 2,6-DNP was measured based on the calibration curve, which is shown in Figure 8b, based on data provided in Table 2. The 2,6-DNP sensor based on the Ag-decorated Chitosan/SrSnO_3_ NC/PEDOT:PSS/GCE was shown to be reliable. Thus, the sensor can potentially be used for the development of microsized electrochemical devices, which are used in situ analyses. 

## 4. Conclusions

In this approach, sol-gel-prepared nanocomposites (NCs) of Ag-decorated Chitosan/SrSnO_3_ show a good structural morphology, which is favorable for the electrochemical detection of 2,6-DNP in a phosphate buffer medium at pH 7.0. The assembled sensor based on the Ag-decorated Chitosan/SrSnO_3_ NC/GCE was used for the detection of 2,6-DNP in a wider range of 1.5~13.5 µM by using a conducting PEDOT:PSS coating binder. In addition to this, the fabricated sensor probe exhibited the highest sensitivity of 54.032 µA µM^−1^ cm^−2^, relatively low LOD of 0.18 ± 0.0088 µM, and good LOQ of 0.545 µM. In addition, it has good reproducibility and a short response time. The 2,6-DNP sensor was applied to analyze a real sample and exhibited significant and reliable performances. Thus, this reliable and easy electrochemical detection method of developing an electrochemical sensor probe has potential environmental applications for the safety of environmental and healthcare fields in a large scale.

## Figures and Tables

**Table 1:** Comparison studies of 2,6-DNP detection based on various nanocomposite materials by electrochemical approaches.

Electrode Materials	LOQ	LDR	Sensitivity	Ref.
PVC (FPVC)	1.00 μM	2.5 µM∼7.5 mM	---	[46]
Mesoporous silica	3.52 μM	3.62~72.4 mM	---	[47]
Cu–BTC/GCE	0.1 μM	0.2~10 μM	15.98 A μM^−1^ cm^−2^	[48]
BaO NR/GCE	0.50 μM	1.5~9.0 μM	17.6 μA μM^−1^ cm^−2^	[49]
CPE	0.04 mM	0.2~10 mM	---	[50]
Ag–Chitosan/SrSnO_3_ NC/GCE	0.18 µM	1.5~13.5 µM	54.03 μA μM^−1^ cm^−2^	This study

**Table 2:** Analysis of real samples with Ag-decorated Chitosan/SrSnO_3_ NC/PEDOT:PSS/GCE sensor probe by electrochemical approach in room conditions.

Real Samples	Added 2,6-DNP Conc. (µM)	Measured 2,6-DNP Conc. ^a^ by Ag-Decorated Chitosan/SrSnO_3_ NCs (µM)			Average Recovery ^b^ (%)	RSD ^c^ (%) (*n* = 3)
		R1	R2	R3		
Underground water	6.00	5.96	5.90	5.92	98.78	0.52
Sea water	6.00	5.92	5.88	5.94	98.56	0.52
Tap water	6.00	5.86	6.06	5.96	99.03	1.68

^a^ Mean of three repeated determination with Ag-decorated Chitosan/SrSnO_3_ NC/GCE. ^b^ Concentration of 2,6-DNP determined/Concentration taken. (Unit: µM). ^c^ Relative standard deviation value indicates precision among three repeated measurements (R1, R2, and R3).

## Data Availability

Data will be available upon reasonable request.
